# Supplementary material for: An Advanced Rider-Cornering-Assistance System for PTW Vehicles Developed Using ML KNN Method
Source: Sensors (Basel). 2023 Jan 31;23(3):1540. doi: 10.3390/s23031540 (PMC9920225; doi:10.3390/s23031540)
Supplement: Supplementary file 1 [file sensors-23-01540-s001.zip › sensors-2173983-supplementary.pdf]

Supplementary

Test	Vehicle	Characteristics of the vehicle			Contact road-tire				Driver behaviour				Referent vehicle
		R_n	R_f (m)	R_r (m)	Ml_{Tire} (ratio)	μ_{lat}	P_{Tire} (ratio)	$Tire_{Type}$ (ratio)	C_{veh}	C_{ct}	$Time$ (ratio)	Ml_{driver} (ratio)	$C_{position}$ (1/m)
P1	HONDA CBR500R/F/X	0.017	0.182	0.201	0.29	0.80	1.0343	0.56	0.7558	0.6817	0.7	0.5	0.01
P2	LAGENDA 115 R6	0.012	0.180	0.178	0.25	0.90	0.5000	0.76	0.8044	1.0988	0.7	0.5	0.02
P3	LAGENDA 115 FZ150	0.019	0.117	0.121	0.42	0.84	0.5000	0.94	0.7200	1.1731	0.7	0.7	0.02
P4	HONDA CBR500R/F/X	0.017	0.180	0.195	0.05	0.44	1.1060	0.26	0.7568	1.4538	0.7	0.8	0.01
P5	HONDA CB650F	0.016	0.202	0.218	0.22	0.57	0.5000	0.26	0.7534	0.6444	0.9	0.8	0.03
P6	LAGENDA 115 XJ6	0.012	0.205	0.212	0.31	0.71	1.2020	0.64	0.8141	0.6418	0.9	0.5	0.05
P7	LAGENDA 115 XV950R BOLT	0.021	0.254	0.239	0.24	0.39	0.5000	0.28	0.6882	0.4077	0.7	0.5	0.05
P8	HONDA CB650F	0.019	0.207	0.210	0.46	0.21	0.5000	0.28	0.7011	1.2042	0.9	0.5	0.01
P9	LAGENDA 115 R1	0.012	0.190	0.190	0.77	0.44	0.5000	0.71	0.8040	0.8822	0.7	0.5	0.01
P10	LAGENDA 115 NMAX 155	0.012	0.125	0.117	0.90	0.56	0.6346	0.91	0.7924	0.0945	0.7	0.5	0.05
P11	HONDA NC750X	0.011	0.223	0.203	0.55	0.56	0.5000	0.73	0.7905	0.4427	0.7	0.5	0.05
P12	LAGENDA 115 R6	0.018	0.195	0.189	0.89	0.95	1.0496	0.77	0.6988	0.2171	0.7	0.5	0.03
P13	HONDA NEW PCX	0.011	0.134	0.138	0.64	0.28	0.7761	0.64	0.8082	0.1591	0.7	0.7	0.05
P14	LAGENDA 115 FZ150	0.012	0.147	0.122	0.49	0.12	1.2406	0.50	0.7817	0.2158	0.7	0.5	0.01
P15	LAGENDA 115 Y15ZR	0.010	0.122	0.105	0.10	0.82	0.6655	0.52	0.7931	1.2216	0.9	0.7	0.01
P16	LAGENDA 115 MT09	0.014	0.183	0.196	0.80	0.51	0.5000	0.82	0.7981	0.0985	0.7	0.8	0.05
P17	HONDA MSX125	0.013	0.116	0.093	0.73	0.51	0.8882	0.16	0.7325	0.6152	0.8	0.5	0.05
P18	LAGENDA 115 LC135	0.017	0.111	0.115	0.25	0.65	1.4923	0.19	0.7084	1.1549	0.7	0.5	0.03
P19	LAGENDA 115 FZ150	0.018	0.118	0.146	0.58	0.03	0.5000	0.63	0.7725	0.1416	0.7	0.5	0.05
P20	HONDA CBR250R	0.015	0.164	0.139	0.28	0.53	0.8175	0.81	0.7178	1.1342	0.8	0.7	0.02
P21	LAGENDA 115 NMAX 155	0.016	0.116	0.124	0.19	0.31	0.9558	0.79	0.7525	2.6715	0.7	0.5	0.01
P22	HONDA CBR1000RR	0.014	0.190	0.200	0.68	0.31	0.5000	0.29	0.7763	0.9908	0.7	0.5	0.01

P23	LAGENDA 115 MT09	0.017	0.185	0.198	0.95	0.53	1.0687	0.40	0.7497	0.5666	0.7	0.9	0.03
P24	LAGENDA 115 FZ150	0.015	0.125	0.141	0.57	0.20	0.7022	0.76	0.8000	0.5664	1.0	0.9	0.01
P25	HONDA CBR1000RR	0.017	0.211	0.205	0.83	0.13	0.5000	0.97	0.7113	0.0625	0.7	0.5	0.05
P26	HONDA NEW PCX	0.013	0.111	0.119	0.59	0.48	1.2013	0.92	0.7820	0.2840	0.9	0.5	0.05
P27	HONDA CB650F	0.010	0.206	0.223	0.31	0.36	0.5000	0.90	0.8506	0.3836	0.7	0.5	0.05
P28	HONDA FUTURE125	0.015	0.120	0.117	0.37	0.02	0.5000	0.11	0.7081	0.3639	0.8	0.8	0.05
P29	HONDA CBR500R/F/X	0.014	0.188	0.205	0.75	0.61	1.0953	0.02	0.7945	0.2961	0.7	0.6	0.05
P30	HONDA CBR250R	0.015	0.167	0.154	0.46	0.68	1.1238	0.41	0.7320	0.5043	0.8	0.7	0.05
P31	LAGENDA 115 NMAX 155	0.012	0.134	0.135	0.76	0.23	1.0223	0.90	0.8085	0.0931	0.7	0.5	0.05
P32	LAGENDA 115 NMAX 155	0.011	0.140	0.142	0.64	0.69	0.5860	1.00	0.8111	0.2745	0.7	0.5	0.05
P33	HONDA CBR250R	0.010	0.139	0.142	0.93	0.67	1.3760	0.13	0.8392	0.6115	0.9	0.9	0.05
P34	LAGENDA 115 MT09	0.010	0.181	0.193	0.36	0.07	0.5000	0.61	0.8520	0.1187	0.7	0.5	0.05
P35	HONDA CBR250R	0.009	0.159	0.166	0.99	0.75	0.5000	0.58	0.8514	0.5283	1.0	0.5	0.05
P36	HONDA NC750X	0.013	0.214	0.217	0.06	0.24	0.5000	0.65	0.7822	0.4491	0.7	0.5	0.05
P37	HONDA NC750X	0.011	0.226	0.208	0.13	0.82	1.0745	0.10	0.8027	1.2132	0.7	0.8	0.01
P38	LAGENDA 115 FZ150	0.011	0.118	0.128	0.18	0.95	1.3799	0.02	0.8500	0.9411	0.7	0.6	0.05
P39	LAGENDA 115 FZ150	0.018	0.139	0.126	0.43	0.06	1.2622	0.77	0.6997	0.1856	0.8	0.5	0.03
P40	LAGENDA 115 MT09	0.011	0.179	0.182	0.35	0.72	0.6535	0.48	0.8361	0.6165	0.7	0.5	0.05
P41	LAGENDA 115 LC135	0.010	0.096	0.102	0.32	0.55	1.1486	0.39	0.8328	0.6313	0.7	0.5	0.05
P42	HONDA CBR500R/F/X	0.016	0.189	0.205	0.35	0.16	0.6511	0.01	0.7713	0.4114	0.7	0.7	0.02
P43	LAGENDA 115 EGO-AVANTIZ	0.012	0.092	0.113	0.47	0.60	0.5000	0.88	0.8556	0.4570	0.7	0.5	0.05
P44	LAGENDA 115 FZ150	0.018	0.139	0.121	0.15	0.24	1.1971	0.11	0.6884	0.4371	0.7	0.5	0.05
P45	HONDA MSX125	0.011	0.087	0.103	0.86	0.56	0.5000	0.72	0.8488	0.2491	0.7	0.5	0.03
P46	HONDA FUTURE125	0.011	0.118	0.093	0.65	0.42	1.2596	0.15	0.7394	0.4785	0.8	0.5	0.05
P47	LAGENDA 115 FZ150	0.015	0.134	0.136	0.75	0.69	0.5000	0.07	0.7709	0.8541	0.8	0.6	0.05
P48	LAGENDA 115 XMAX 250	0.015	0.196	0.208	0.42	0.84	0.8418	0.28	0.7297	0.8152	0.7	0.6	0.05

P49	LAGENDA 115 NMAX 155	0.011	0.116	0.118	0.39	0.51	0.5000	0.86	0.8134	0.4478	0.7	0.7	0.05
P50	LAGENDA 115 LC135	0.012	0.115	0.120	0.10	0.11	0.5000	0.13	0.7922	1.1535	0.7	0.5	0.03
P51	LAGENDA 115 XV950R BOLT	0.019	0.256	0.236	0.79	0.39	1.3705	0.47	0.7171	0.9403	0.7	0.5	0.05
P52	LAGENDA 115 MT09	0.013	0.178	0.184	0.01	0.42	1.3366	0.60	0.8053	0.4995	0.7	0.5	0.05
P53	HONDA CB650F	0.012	0.204	0.201	0.38	0.29	1.0405	0.89	0.7993	0.3686	0.8	0.9	0.03
P54	LAGENDA 115 R1	0.017	0.185	0.197	0.02	0.05	0.5543	0.32	0.7430	0.2346	0.7	1.0	0.05
P55	HONDA CB650F	0.015	0.202	0.198	0.49	0.98	0.9547	0.96	0.7420	0.3527	0.8	0.9	0.05
P56	LAGENDA 115 MT09	0.012	0.182	0.181	0.03	0.13	1.4416	0.83	0.8154	0.2821	0.7	0.5	0.05
P57	HONDA CBR500R/F/X	0.014	0.196	0.197	0.41	0.01	0.7638	0.61	0.7728	0.0655	0.8	0.5	0.05
P58	HONDA NC750X	0.017	0.218	0.200	0.12	0.06	0.8273	0.71	0.6860	0.2023	0.8	0.5	0.05
P59	LAGENDA 115 XJ6	0.012	0.212	0.211	0.34	0.33	0.5000	0.82	0.8027	0.4170	0.7	0.8	0.05
P60	LAGENDA 115 LC135	0.016	0.105	0.118	0.44	0.41	1.2225	0.15	0.7522	0.3851	0.7	0.8	0.05
P61	LAGENDA 115 XMAX 250	0.012	0.201	0.195	0.73	0.24	0.5000	0.66	0.7630	0.2568	0.7	1.0	0.05
P62	LAGENDA 115 XV950R BOLT	0.019	0.259	0.250	0.71	0.50	1.0821	0.04	0.7279	0.6924	0.7	0.8	0.05
P63	HONDA NSS300	0.016	0.195	0.182	0.74	0.90	0.5000	0.36	0.6863	2.4830	0.7	0.5	0.03
P64	HONDA CBR1000RR	0.012	0.207	0.215	0.39	0.97	0.5000	0.31	0.8168	0.8554	0.7	0.6	0.05
P65	LAGENDA 115 R6	0.014	0.202	0.179	0.06	0.91	0.6340	0.48	0.7503	0.8359	0.7	0.5	0.05
P66	LAGENDA 115 R6	0.010	0.205	0.193	0.85	0.15	0.5000	0.94	0.8322	0.0581	0.8	0.5	0.05
